# Good clinical practice advice for the management of patients with gynaecological cancer during the COVID-19 pandemic in Nigeria and other resource-constrained countries

**DOI:** 10.3332/ecancer.2020.1075

**Published:** 2020-07-17

**Authors:** Kehinde S Okunade, Adeyemi A Okunowo, Ephraim O Ohazurike, Rose I Anorlu

**Affiliations:** 1 Department of Obstetrics and Gynaecology, Faculty of Clinical Sciences, College of Medicine, University of Lagos, PMB 12003, Idi-Araba, Lagos, Nigeria; 2 Department of Obstetrics and Gynaecology, Lagos University Teaching Hospital, PMB 12003, Idi-Araba, Lagos, Nigeria; a https://orcid.org/0000-0002-0957-7389

**Keywords:** COVID-19, cancer treatment, coronavirus, gynaecological oncology, Nigeria

## Abstract

The impact of the COVID-19 pandemic on healthcare services in settings with under-resourced health systems such as that of Nigeria is likely to be substantial in the coming months. The gynaecological oncology services still need to be prioritised as an essential core health service. There are increasing concerns from both physicians and patients regarding how to manage patients diagnosed with cancer during this pandemic as evidence suggests a substantial increase in the risk of COVID-19-related deaths amongst patients with cancer. However, we recognise that despite this great challenge, we must continue to provide the highest quality of care to the patients, whereas, at the same time, ensure adequate safety not only for the patients and their families but also for the entire oncology team. We advocate that due to the widespread travel restrictions and inability to refer patients for the highest level of care at this period, centres without radiotherapy facilities as seen in most resource-limited settings should always consider lower level care options such as the use of chemotherapy pending when there is a better access to these facilities. We, therefore, developed this good clinical practice advice to staff of the gynaecological oncology unit in the centre and other resource-constrained settings for the management of patients with gynaecological cancer during the COVID-19 pandemic.

## Introduction

In January 2020, the current novel coronavirus disease 2019 (COVID-19) outbreak was declared as a Public Health Emergency of International Concern by World Health Organisation [1]. Most of the countries around the world including Nigeria have seen the cases of COVID-19, and many are experiencing outbreaks with widespread community transmission [2]. As at the time of writing this guideline, most of the states in Nigeria have already reported a widespread community transmission of COVID-19 [3]. The world is currently faced with an unprecedented crisis as the pandemic spreads across different nations. As a result, many countries are now taking steps to implement strategies to contain the spread of the virus. The healthcare system of most countries all over the world is overburdened with the huge task of managing the COVID-19 pandemic. As a result, Nigeria and other LMICs with very frail healthcare systems characterised by low funding, inadequate manpower and poor infrastructures may collapse under the weight of the pandemic if no drastic measures are put in place to contain the spread of the virus and, at the same time, manage non-COVID-19-related health problems such as cancer. There are increasing concerns by both physicians and patients regarding how to manage patients diagnosed with cancer during this pandemic as early reports suggest a substantial increase in the risk of COVID-19-related deaths amongst patients with cancer. This risk is, perhaps, the highest amongst patients older than 60 years and those with pulmonary compromise [4, 5]. We recognise that during this challenging period, we must continue to provide the highest quality of care to the patients with gynaecological cancer, whereas, at the same time, ensure adequate safety not only for the patients and their families but also for the entire oncology team [6]. We must weigh the risk of patients contacting COVID-19 and dying from it during their treatment against the risk of cancer progressing or causing death if it is not treated appropriately [7]. To this end, this good clinical practice advice was developed by the Gynaecological Oncology Unit of the Lagos University Teaching Hospital to propose the strategies to optimise the care of patients with gynaecological cancer in centres with recognised gynaecological oncology units in Nigeria. It will also offer potential options to alleviate the burden to the fragile healthcare system in the resource-constrained setting, especially when available resources may need to be diverted to the direct care of patients affected by COVID-19. The practice advice is intended to be a dynamic tool to guide care and certainly not as a strategy for permanent change in the patterns of practice. The situation with COVID-19 is evolving daily, and the recommendations in this clinical practice advice will continue to be updated if and when new evidence or information becomes available.

## General principles

- In considering the management of disease, we must consider options that offer the patients a treatment plan that addresses their disease, whereas, at the same time, limit their risk of exposure to COVID-19, bearing in mind the constraints and limitations in resource-constrained settings like ours.
- Since access to radiotherapy facilities is limited in most of the treatment centres and because of the need to reduce major surgical procedures during this pandemic, the use of chemotherapy should be encouraged as a feasible alternative to care as most of the centres in resource-constrained countries have access to chemotherapy.
- Clinicians may need to prioritise treatment for patients who need it most and all decisions must be taken with the multidisciplinary team (MDT) input, and these should be communicated clearly to the patients [6].
- It is now imperative that we explore options that reduce the number of procedures or surgical interventions that may be associated with prolonged operative time, risk of major blood loss necessitating transfusion with blood products, risk of infection to the medical personnel or admission to the intensive care unit (ICU) [8].
- Consider postponing any type of intervention that is not absolutely necessary, such as routine imaging studies or serum markers, in patients who are asymptomatic and have no evidence of disease based on the most recent evaluation [8].
- Patients and their families should be fully involved in discussions on whether the risks of beginning or continuing their cancer treatment could outweigh the benefits, given that evidence from studies suggests that patients with cancer are more susceptible to COVID-19 infection [6] because of their systemic immunosuppressive state caused by the malignancy and systemic anticancer treatments [9–13].
- In particular, where patients are considered at high risk (e.g. due to a combination of age, performance status, comorbidities, cancer burden and frailty), an individualised decision must be made with full patient involvement, to understand the potential pros and cons of anticancer treatment versus delaying definitive treatment during COVID-19 pandemic [6]. The need for perioperative intensive care support should be incorporated into any decision-making processes, due to the high risk of such support not being available due to emergency care requirements for COVID-19 patients [8].
- The increased morbidity and mortality risks from a potential COVID-19 infection caused by embarking on cancer surgery or commencing anticancer treatments during the pandemic and the options of deferring surgery or non-surgical treatments should be included as part of the consent process and clearly documented [6].
- Where conservative methods of treatment have been shown to have efficacy, e.g. levonorgestrel intrauterine system (LNG-IUS) for early-stage uterine cancer in patients with comorbidities, elderly or unfit for treatment, these should be considered and discussed with patients [6, 8].
- Gynaecological oncologists should adopt greater utilisation of non-surgical options including radiotherapy when available or chemotherapy to delay major resection surgery until there is greater availability of services, such as ICU support [6].
- The use of granulocyte‑colony-stimulating factor prophylaxis, where available and affordable, should be more liberal than usual to avoid the risk and decrease the duration of immunosuppression [14].
- In the case of patients developing pneumonia during treatment, COVID‑19 must be considered as one of the differentials.
- All healthcare workers in the gynaecological oncology team should wear appropriate personal protective equipment (PPE) (see Table 1) [14].
- The gynaecological oncology team should be divided into three groups, with each working for 1 week at a time. This will ensure that in the case of any exposure, only members of the affected group will be isolated/quarantined, whereas the other groups will continue to render uninterrupted services [15].
- All patients and their caregivers should be provided with the unit’s telephone number to enable them to contact any designated member of the healthcare team when necessary.

## Outpatient clinic visits

- Face-to-face visits to the hospital should be minimised as much as possible, and the alternatives for routine follow-up visits, such as the use of telehealth (telephone or video-consultation) if available, should be considered.
- Limit the number of healthcare providers (physicians and oncology nurses) involved in providing ambulatory care to minimise exposure to all involved. The unit may be further divided into groups with each headed by a specialist trained in the management of gynaecological cancer.
- The hospital or unit should triage patients and their accompanying persons at the entry points and allow entry to only those with a low risk of COVID‑19 and those without COVID‑related symptoms. Manage patients with a high risk of COVID-19 as shown in Table 2.
- Limit the number of accompanying family members to only one person.
- All patients and their accompanying persons should be encouraged to put on a face mask before entering the hospital premises. Where surgical face masks are not available, the use of a ready-made clean piece of cloth to cover the nose and mouth will suffice.
- Patients with existing follow-up appointments are encouraged to notify the healthcare team of any new or concerning issues by telephone before their visit. Where this is not feasible, this information should be immediately volunteered at the point of entry and triaging.
- Routine face-to-face appointments to the gynaecological oncology unit for patients with suspected or confirmed COVID-19, who are self-isolating at home, should be delayed until after the recommended period of self-isolation or full recovery after treatment.
- Use of personnel protection remedies (such as the use of facemasks and physical distancing) and social isolation should be reinforced for cancer patients to reduce the probability of contracting infection [16].

## Patients categorisation for treatment

- Treatment in cancer cases is time sensitive [7].
- The decision to commence treatment should be made based on the type and stage of the disease, medical condition of the patient, COVID-19-associated risks and available logistic support including adjuvant treatment services such as radiotherapy and chemotherapy [7].
- Patients with gynaecological cancer should be triaged for observation or intervention. The basic principle is to prioritise intervention for oncological emergencies, initiate treatment for aggressive or advanced-stage disease and reasonably postpone intervention for benign, pre-invasive or early-stage low-grade malignancy after informed consent. To carry out surgical procedures in an orderly and safe manner, an acuity scale is suggested to guide gynaecological oncologists whether to postpone or perform surgical procedures during the COVID-19 pandemic [15, 17].

### Category 1 (low acuity surgery)

This includes conditions that are not life-threatening (pre-invasive lesion of cervix or endometrium) and for which surgery can be postponed for a few weeks or months.

### Category 2 (intermediate acuity surgery)

This includes conditions that are not life-threatening but with potential for future morbidity or mortality, e.g. early-stage cervical cancer and well-differentiated endometrial cancers with co-morbidities. In case surgery is done for patients in this category, they should be considered for early postoperative discharge.

### Category 3 (high acuity surgery)

This includes life-threatening conditions, e.g. highly symptomatic patients, type II endometrial cancers, ovarian cancer, interval debulking surgery after 3–4 cycles of chemotherapy, uterine sarcoma, those in need of emergency procedures, resection of recurrent tumours and gestational trophoblastic neoplasia. Surgery should not be postponed if a patient is low risk for COVID-19 and resources permit. An MDT discussion and planning of therapy are preferred before surgery for realistic expectations. Only carefully selected cases should be subjected to neoadjuvant therapy after adequate counselling of patients and their caregivers.

## Inpatient care

For patients who require inpatient treatment (surgery or systematic therapy), strategies to prevent COVID-19 transmission during hospitalisation should be adopted and these include [17, 18]:

- Pre-hospitalisation screening of all cancer patients using the checklist in Table 2 and confirmation test using the reverse transcriptase-polymerase chain reaction (RT-PCR) test for COVID-19 in patients suspected of having the infection. In settings with low or no testing capacity, patients with suspected COVID-19 who are not critically ill should be encouraged to self-isolate at home for a minimum of 14 days.
- Patients with confirmed COVID-19 should be isolated from other patients (‘cohorting’)
- Shift elective urgent inpatient diagnostic and minor surgical procedures to outpatient settings, when feasible.
- Restriction of visitors
- Physical distancing
- Daily staff controls by minimising the number of staff attending to the inpatients at each particular time
- Adoption of measures to decrease hospital stay—hospitalisation is mostly dependent on peri-operative management. Thus, evidence-based protocols focusing on the surgical management of cancer patients should be adopted, i.e., the enhanced recovery after surgery (ERAS) protocols [19–21].

## Surgical procedures

- The first consideration should be given to surgery that is unlikely to require adjuvant treatment [15].
- Cases who are likely to require ICU care are best postponed until when feasible as these facilities may be unavailable for routine cancer cases during the COVID-19 pandemic [7].
- In an advanced disease where treatment is only palliative, surgery is best postponed. Patients should be considered for chemotherapy if appropriate.
- Until such time that COVID-19 testing becomes widely available in the resource-limited settings, all surgical cases should be managed as if they were COVID-19 positive unless proven otherwise, and all necessary precautions and protective measures should be taken in the operation theatre to protect the healthcare workers as well as other patients.
- If available, the RT-PCR test for COVID-19 should be done at most 7 days before admission for surgery [22].
- Where the capacity for COVID-19 RT-PCR testing is not available, checking for symptoms such as fever, cough, easy fatiguability, difficulty in breathing, sore throats or flu-like symptoms should be done at the time of admission [22] (see checklist in Table 2).
- If COVID-19 testing is not possible before surgery or in case an emergency procedure has to be performed, surgeons should wear the appropriate PPE and take all other infection prevention and control measures.
- Cancer surgery should be done at ‘clean sites’ that are separate from units managing patients with suspected/confirmed COVID-19. Sharing of resources such as theatre and ward capacity with units managing COVID-19 patients should be discouraged.
- After surgery, ERAS protocols may be implemented for rapid recovery, reduced postoperative complications (including respiratory complications), early discharge and decrease the chance of being infected with COVID-19 [20]. The key components of ERAS protocols in the preoperative setting are shown in Table 3.

## Disease-site specific recommendations

### Cervical cancer

It is recommended to postpone the diagnostic evaluation and treatment of individuals with low-grade cervical screening tests for 6–12 months and individuals with high-grade cervical screening tests for 3 months [8]. For invasive cervical tumours, radiotherapy and concomitant chemoradiation can be done in place of surgery as first-line treatment whenever possible, and this should be started within 4 weeks [15]. Low-risk invasive lesions (<2 cm, favourable histologies) may be considered for standard treatment and, in places with limited access to surgery, considered conisation or simple trachelectomy and reassess in 3 months. For bulky lesions (>2 cm), consider chemoradiation if available or neoadjuvant chemotherapy in setting without radiotherapy facilities. Non-resectable and advanced cases should follow standard treatment with the suggestion of hypofractionation of radiotherapy doses (to decrease hospital visits) [8, 23]. Palliative treatment in recurrent cervical cancer can be offered in single‑fraction radiation schedules. However, due to the widespread travel restrictions and inability to refer patients for the highest level of care, centres without radiotherapy facilities, as seen in most resource-limited settings, should always consider a lower level care options such as the use of chemotherapy pending when there is a better access to these facilities for the patients [24]. Supportive treatment such as the use of analgesics and antibiotics should be considered for symptomatic management of patients with advanced diseases.

### Ovarian cancer

In early‑stage ovarian cancer, staging laparotomy should not be delayed, and adjuvant treatment should be offered with platinum-based chemotherapy. For advanced disease, neoadjuvant therapy should be given, and interval cytoreduction should be performed after 3–6 cycles. If recurrent cases are symptomatic, only then systemic therapy should be offered [14]. Single‑agent therapy or metronomic combination should be preferred [8, 23].

### Endometrial cancer

Perform outpatient endometrial biopsies only for patients with high suspicion of endometrial cancer. For low-risk patients (type 1 histology) and grade 1 tumours, consider non-surgical therapies such as hormone therapy and LNG-IUS insertion and may postpone treatment for 1–2 months. For intermediate and high risks (type 2 histology and grade 2/3), consider performing hysterectomy with salpingo-oophorectomy, omentectomy and pelvic lymph node dissection. Adjuvant chemoradiation can be deferred in high‑risk cases and omitted for patients with low and intermediate risk [14]. In advanced disease, either progesterone or radical radiation with brachytherapy can be administered. In inoperable cases, palliative therapy with chemotherapy using carboplatin–paclitaxel should be offered [8, 23].

### Vulvar cancer

For early-stage tumour, surgery can be avoided for 4–6 weeks, if possible. Radiotherapy and chemotherapy treatments should be considered in locally advanced diseases that require extensive resections and the need for flap rotation. In the presence of metastatic disease, systemic therapy can be considered in the presence of symptoms [8, 23]. For unresectable locally advanced or metastatic disease, consider palliative treatment in the form of single‑fraction radiation therapy if available, or otherwise, the use of single-agent chemotherapy such as Cisplatin may be adopted.

### Gestational trophoblastic tumours

Due to the excellent potential to achieve a cure and a high chance of metastases at diagnosis, immediate systemic therapy is required [23, 25, 26]. Patients with low-risk (FIGO score ≤6) and high-risk (FIGO score >6) trophoblastic tumours should be administered the standard single- and multi-agent regimens, respectively, in an outpatient setting.

## Treatment for patients with suspected or confirmed COVID-19

- All suspected cases should be tested to exclude COVID-19 infection before instituting any systemic treatment.
- As cancer patients are more likely than the general population to contract COVID-19 and with often fatal outcome [27], their surgical management and all other oncological treatment should be postponed for at least 14 days to enable them to complete treatment or make full recovery from the virus.
- For those patients in whom treatment cannot be postponed, i.e., bowel perforation, peritonitis, fistulation, anastomotic leak, intestinal or urinary obstruction, severe pelvic bleeding and abscess, the managing team should use PPE and take necessary precautions as per the hospital protection guidance.
- Identify and train dedicated staff to care for COVID-19-positive cancer patients
- The patient should be received at the entrance of the isolation unit by staff wearing appropriate PPE and be provided with a fluid-resistant surgical mask. The woman’s face mask should not be removed until she is isolated and alone in a suitable room or cohort bay.
- During surgical procedures, the number of staff in the operating theatre should be kept to a minimum, and all must wear appropriate PPE.
- All staff should be trained on infection prevention and control, and the use of PPE so that 24-hour emergency surgeries are available and possible delays is reduced.
- There should be regular dry-run simulation exercises to prepare staff, build confidence and identify areas of concern to prepare for emergency transfers to the operating theatre.

## Equipment disinfection

- PPE should be provided to housekeepers/cleaners for use in the proper disinfection of the outpatient clinic space and operating theatre, especially if used for the care of a suspected or confirmed COVID-19 case.
- All used equipment and instruments must be promptly disinfected by soaking them in chemical disinfectants such as chlorine or by autoclaving. Used PPE must be properly removed and carefully disposed of following the hospital protocol.

## Post-treatment follow-up

- Post-therapeutic face-to-face follow-up consultations should be postponed for 1–2 months if possible.
- Follow-up can be performed by teleconsultation, if available. However, follow-up of endometrial and/or cervical cancer is based mainly on clinical examination, and this cannot be replaced by teleconsultation or follow-up consultation after 1–2 months.

## Conclusion

This document highlights practical advice for the management of patients with gynaecological cancer during the COVID-19 pandemic in Nigeria with applicability in similar resource-constrained settings in the world. In the management of patients with gynaecological cancer, we must consider options that offer the patients a treatment plan that addresses their disease, whereas, at the same time, limiting their risk of exposure to COVID-19. It is now imperative to postpone any type of intervention that is not an emergency or explore options that reduce the number of procedures or surgical interventions that may be associated with prolonged operative time, risk of major blood loss necessitating transfusion with blood products, risk of infection to the medical personnel or admission to the ICU. There should be greater utilisation of non-surgical options including chemotherapy and radiotherapy if available during the period of this pandemic to delay major resection surgery until there is a greater availability of services, such as ICU support. Since cancer patients are more likely than the general population to contract COVID-19 and often with fatal outcome, their surgical management and all other oncological treatment should be postponed for at least 14 days, if possible, to enable them to complete treatment or make full recovery from the virus.

## Competing interest

The authors declared no conflicts of interest.

## Funding declaration

KSO had partial time protection for this write-up through the Conquer Cancer International Innovation Grant under Project ID 16576.

## Figures and Tables

**Table 1:** Appropriate personal protective equipment (PPE) for staff during COVID-19 pandemic*

The minimum PPE to attend to any patient during COVID-19 pandemic should consist of: Sterile gloves and surgical masks with visors or face shields for non-surgical cases; and For surgical procedures, the surgeons will also require the following PPE: The above-elbow length gloves N-95 face masks and A full water-resistant disposable gown The minimum PPE to attend to any suspected or confirmed COVID-19 patient should consist of: A full water-resistant disposable gown Sterile gloves and surgical masks with visors or face shields for non-surgical cases; and For surgical procedures, the surgeons will also require the following PPE: The above-elbow length gloves N-95 face masks and Tyvek disposable Hazmat coverall suits**.

* Adapted from “Recommended PPE for healthcare workers by secondary care inpatient clinical setting, NHS and independent sector by Public Health England (PHE)”.

** Advisable to be used particularly in hospitals where there are no negative pressure surgical theatres or rooms.

**Table 2:** Checklist for assessing patient’s risk of COVID-19*

Symptoms	Point systems
Cough	1
Catarrh/running nose	1
Sore throat	1
Diarrhoea Loss of taste or smell	1 1
Myalgia/ Body pains	1
Headaches	1
Fever (Temperature ≥37.8^O^C)	1
Difficulty in breathing	2
Easy fatiguability	2
Contact with an individual who tested positive to COVID-19	3

* Adapted from the Lagos University Teaching Hospital COVID-19 screening instrument for triage, April 2020.

**Score results**
0–2: No action required. Allow into the clinic area. 3–5: If patient has no cough or breathlessness. No additional action required. Allow to clinic area If patient has either cough or breathlessness. Isolate and request for an urgent Chest X-ray 6–15: Suspicious of COVID-19. Isolate and invite the Infectious Disease Unit to review.

**Table 3:** Enhanced Recovery After Surgery (ERAS) protocols*

Items
No mechanical bowel preparation
Patients may eat a light meal up until 6 hours, and consume clear fluids including oral carbohydrate drinks up until 2 hours, before initiation of anaesthesia
Use of pre-medications (acetaminophen, nonsteroidal anti-inflammatory drugs, anti-emetics)
Maintenance of normothermia and euvolaemia intra-operatively
Avoidance of surgical drains and nasogastric tubes
Infiltration of wound with local anaesthetic
Post-operative nausea and vomiting prophylaxis using ≥2 anti-emetics (multimodal approach)
Early introduction of solid diet post-operatively (day 0–1)
Multimodal narcotic-sparing post-operative analgesia (use of scheduled non-narcotic medications with oral narcotic medications only as needed)
Peripheral lock intravenous when the patient has 600mL oral intake
Remove urinary catheter on postoperative day 1 in the absence of contraindications
Active mobilization

* Adapted from “Guidelines for Perioperative Care in Gynecologic/Oncology: Enhanced Recovery After Surgery (ERAS) Society recommendations – 2019 Update by Nelson G et al [18].
